# Effect of TCF7L2 polymorphism on pancreatic hormones after exenatide in type 2 diabetes

**DOI:** 10.1186/s13098-019-0401-6

**Published:** 2019-01-25

**Authors:** Mari Cassol Ferreira, Maria Elizabeth Rossi da Silva, Rosa Tsuneshiro Fukui, Maria do Carmo Arruda-Marques, Salman Azhar, Rosa Ferreira dos Santos

**Affiliations:** 1 School of Medicine, Unochapeco University, Chapeco, Santa Catarina Brazil; 2 School of Medicine, University of Sao Paulo, São Paulo, Brazil; 3 LIM 18, School of Medicine, University of Sao Paulo, São Paulo, Brazil; 4 Stanford School of Medicine, Palo Alto, CA USA

**Keywords:** Postprandial period, Glucagon-like peptide 1, Polymorphism, Single nucleotide, Proinsulin, Insulin, Insulin resistance, C-peptide

## Abstract

**Background:**

Glucagon-like peptide 1 (GLP-1) stimulates insulin secretion and reduces blood glucose in type 2 diabetes mellitus (T2DM). *TCF7L2* rs7903146 polymorphism has been associated with decreased insulin secretion, reduced GLP-1 action, and possible impaired peripheral insulin sensitivity.

**Objectives:**

To evaluate the postprandial pancreatic hormone response in patients with T2DM carriers of the *TCF7L2* variant rs7903146 (CT/TT) compared with noncarriers of this variant (CC) after treatment with the GLP-1 agonist exenatide.

**Methods:**

Intervention study. Patients with T2DM (*n *= 162) were genotyped for the *TCF7L2* rs7903146 single nucleotide polymorphism (SNP). Individuals with CT/TT and CC genotypes were compared regarding basal serum levels of glucose, glycosylated hemoglobin A1C (HbA1c), HDL, uric acid, insulin, and C-peptide. A subset of 56 individuals was evaluated during a 500-calorie mixed-meal test with measurements of glucose, insulin, proinsulin, C-peptide and glucagon before and after treatment with exenatide for 8 weeks.

**Results:**

Patients with genotypes CC and CT/TT presented similar glucose area under the curve (AUC) 0–180 min before treatment and a similar decrease after treatment (p < 0.001). Before exenatide, insulin levels at 30–120 min were higher in CT/TT versus CC subjects (p < 0.05). After treatment with exenatide, only CT/TT individuals demonstrated insulin reduction at 30–180 min during the meal test (p < 0.05). Patients with the CC genotype presented no differences in insulin concentrations before and after treatment. The areas under the glucagon curve between 0 and 180 min were similar before treatment and reduced after treatment in both groups (p < 0.001).

**Conclusions:**

The presence of the *TCF7L2* rs7903146 T allele in patients with T2DM was associated with increased secretion of insulin response to a mixed-meal test. Furthermore, after treatment with exenatide, only the carriers of the T allele showed significantly decreased postprandial plasma insulin peak levels comparing with non carriers.

## Introduction

Modern genetic analysis is unquestionably one of the most powerful research tools in complex diseases such as diabetes. The canonical signaling pathway plays an important role in the modulation of cell proliferation and survival [25]. In the intestine, this pathway regulates the expression of the proglucagon gene and consequent secretion of glucagon-like peptide 1 (GLP-1) by intestinal endocrine cells in response to meals [11]. GLP-1 is a potent stimulant of insulin secretion and reduces blood glucose levels [7] and GLP-1 administration has also been shown to improve defects in glucose uptake independent of insulin [18]. According to some authors, GLP-1 presents insulin-independent effects in reducing hepatic glucose production [19, 20].

Potential mechanisms by which *TCF7L2* variants influence T2DM include defective insulin secretion [1, 16, 27], deficient insulin processing [33], and decreased sensitivity to GLP-1 [17]. In tissues that are a target for insulin action, GLP-1 is believed to interact with its receptor on the cell membrane and stimulate the synthesis and activation of proteins participating in the intracellular signaling cascade for insulin [2, 19]. Thus, the presence of polymorphic variants of *TCF7L2* would determine a lower GLP-1 effect on target cells, which could also be involved in the regulation of peripheral insulin sensitivity [21]. In addiction, it is questionable whether the presence of these variants would determine a functional defect in GLP-1 signaling in beta cells, as well as in liver and skeletal muscle tissues.

Considering these observations, we propose the possibility that increasing incretin levels through pharmacotherapy might overcome incretin resistance due to genetic variation in *TCF7L2.* Therefore, the objective of the present study was to evaluate whether individuals with T2DM carriers and noncarriers of the polymorphic variant rs7903146 of the *TCF7L2* gene present different postprandial pancreatic hormonal responses after treatment with exenatide.

## Materials and methods

This study was carried out according to the ethical principles of the Declaration of Helsinki. Written consent was obtained from all patients before the start of the procedures. The study protocol was approved by the Ethics Committee for Analysis of Research Projects of the Clinical Hospital at the School of Medicine of the University of São Paulo (HC-FMUSP) (CAPPesq0261/09). Exenatide (Byetta, Eli Lilly do Brasil Ltda., São Paulo, SP, Brazil) was acquired with financial resources from the project.

We selected 182 individuals with T2DM with disease duration < 10 years, body mass index (BMI) between 25 and 35 kg/m^2^, aged 45 to 65 years, and nonusers of insulin, DPP-4 inhibitors, or GLP-1 mimetics. The participants were recruited from the endocrinology outpatient clinic at HC-FMUSP and from campaigns celebrating the World Diabetes Day. After the exclusion of patients with nephropathy, liver disease, and severe heart disease, 162 patients remained in the study. Weight, height, and neck and thigh circumferences were measured in all patients, and BMI and neck/thigh ratios were calculated from the measurements. Peripheral blood was collected for DNA extraction and biochemical and hormonal evaluations. All patients were genotyped for analysis of the presence of the polymorphic variant rs7903146 of the *TCF7L2* gene.

Based on genotyping results, we recruited 56 patients from the initial cohort for a 500-kcal mixed meal test and treatment with exenatide. The patients in this subcohort were paired in regards to duration of diabetes and BMI and were further subdivided into groups CC (n = 26) and CT/TT (n = 30). Subsequent analysis showed that this subcohort was representative of the overall cohort.

### Evaluation of biochemical, hormonal, and inflammatory factors

We used a specific enzymatic-colorimetric method (glucose oxidase–peroxidase, GOD-ANA, Labtest, Lagoa Santa, MG, Brazil) for measurement of plasma glucose. Serum insulin and proinsulin levels were determined by liquid phase and double antibody radioimmunoassay (HI-14K and HPI-15K, respectively, EMD Millipore, Billerica, MA, USA). The samples were centrifuged under refrigeration to preserve activity and then stored at − 20 °C.

Blood samples for measurement of glucagon levels were collected in Vacutainer^®^ tubes (BD, São Paulo, SP, Brazil) containing EDTA and aprotinin (A6279, Sigma-Aldrich, St. Louis, MO, USA) as a protease inhibitor and maintained on ice until final processing. The plasma aliquots were stored at − 20 °C until measurement by liquid phase and double antibody radioimmunoassay (GL-32K, EMD Millipore, Billerica, MA, USA). Serum C-peptide was measured with a radioimmunoassay kit (HCP-20K, EMD Millipore, Billerica, MA, USA).

Laboratory analysis performed before and after exenatide treatment included measurement of C-reactive protein (CRP) and tumor necrosis factor alpha (TNF-α), considering that these cytokines present proinflammatory actions that may interfere with insulin resistance. Measurement of CRP was performed by the ELISA method (DCRP00, human C-reactive protein Quantikine ELISA kit, R&D Systems, Bio-Techne Corporation, Minneapolis, MN, USA) and measurement of TNF-α was done by specific and high sensitivity ELISA (HSTA00D, Human TNF-alpha Quantikine HS ELISA, R&D Systems, Bio-Techne Corporation, Minneapolis, MN, USA).

Serum levels of uric acid and HDL-cholesterol were measured using commercial kits. Serum levels of glycosylated hemoglobin (HbA1c) were determined by high-performance liquid chromatography (HPLC).

### Peripheral blood DNA extraction and *TCF7L2* genotyping

For analysis of genomic DNA, 10 to 12 mL of peripheral blood were collected in three 4 mL Vacutainer^®^ tubes (BD, São Paulo, SP, Brazil) containing 7.2 mg of EDTA and evaluated by salting out. Genotyping of the rs7903146 polymorphism variant of the *TCF7L2* gene was performed by real-time polymerase chain reaction (RT-PCR) using TaqMan fluorogenic 5′ nuclease assay (Applied Biosystems, Foster City, CA, USA). The final predicted volume of RT-PCR was 10 μL, with 5 μL of genomic DNA at 10 ng/μL and 5 μL of TaqMan PCR MasterMix with primers at the ratio of 9:1, as recommended by the manufacturer (assay ID: C29347861_10 for rs7903146). The equipment used was StepOnePlus (Applied Biosystems, Foster City, CA, USA).

### 500-kcal meal test and treatment with exenatide

After fasting for 12 h, a subgroup of 56 patients received a 500-kcal breakfast comprising 50% carbohydrates, 30% proteins, and 20% fat. Blood samples were collected from a forearm vein at 0, 15, 30, 45, 60, 90, 120, 180, and 240 min. Glucose, insulin, proinsulin, C-peptide, and glucagon levels were measured at all time points. TNF-α, CRP, and HbA1c were also measured at 0 min.

The participants in the meal test arm of the study received treatment with exenatide for 8 weeks at a dose of 5 μg twice a day at weeks 1 to 4 and 10 μg twice daily at weeks 5 to 8. On the day of the post-treatment meal test, the patients received a final 10 μg dose of exenatide 30 min before the meal. We also analyzed the participants’ weight, HbA1c, CRP, and TNF-α before and after the exenatide treatment and calculated their insulinogenic index (IGI 30), and homeostasis model assessment (HOMA) index. The IGI 30 evaluates the beta cell function using the first phase of the postprandial insulin response, which in this study was defined as the rate of plasma insulin increase 30 min after the meal: IGI 30 = (I30 − I0)/(G30 − G0). The HOMA index was calculated from plasma glucose and C-peptide levels and was used to estimated beta cell function (%B), insulin sensitivity (%S), and insulin resistance before and after treatment with exenatide.

### Statistical analysis

Genotypes are described as absolute and relative frequencies. The analysis of the Hardy–Weinberg equilibrium for genotype distribution was estimated by the Chi square test. The nominal characteristics of the patients were described according to each genotype and the presence of association among the genotypes, and the characteristics were also verified with the Chi square test or, when the samples were insufficient, with the likelihood ratio test.

Results of laboratory tests and anthropometric characteristics are described according to each genotype and were compared among the genotypes using analysis of variance (ANOVA) or Kruskal–Wallis test. For the SNP rs7903146, we evaluated the CC, CT, and TT genotypes separately and then the CT and TT genotypes as a group and described the results across the categories using Student’s *t* test or Mann–Whitney test.

The analyses of the meal tests were described according to groups (CC and CT/TT), time points (pretreatment and post-treatment), and moment of evaluation in the curve (test times) using summary measures, followed by comparisons between groups, time points, and moments with three-factor ANOVA with repeated measures for moment and time point factors. A symmetrical component matrix was applied in the evaluation time points and moments. For measurements presenting statistically significant differences, we applied Bonferroni multiple comparisons or contrasts to verify among which groups, time points, or moments the differences occurred and to estimate them. We compared the groups and moments at each time point in the follow-up curve using Scheffé contrasts for multiple comparisons. For the areas under the curves (AUCs) and IGI 30, we used two-way ANOVA and repeated measures between the moments of evaluation, with a symmetrical component matrix between the moments, followed by Bonferroni multiple comparison.

Weight, HbA1c, CRP, and TNF-α before and after treatment were compared according to the genotypes (CC and CT/TT), using generalized estimating equations (GEE), with weight and HbA1c using normal distribution and binding function identity, whereas, for CRP and TNF-α, gamma distribution and identity link function were considered. All analyses were followed by Bonferroni multiple comparisons to identify between which genotypes or moments the differences in the parameters occurred.

The AUCs were generated with GraphPad Prism (GraphPad Software Inc., San Diego, CA, USA) and the other statistical analyses were performed with IBM SPSS, version 17 (IBM Corp, Armonk, NY, USA) and SAS, version 9.1 (SAS Institute Inc., Cary, NC, USA). Mean results and respective standard errors are illustrated with graphs. The tests were performed with a significance level of 5%.

## Results

### Baseline clinical and laboratory variables

The mean age of the overall cohort was 57.1 ± 7.3 years. The prevalences of the CC, CT, and TT *TCF7L2* genotypes were 41.4%, 47.5%, and 11.1%, respectively. Both groups did not differ in regards to diabetes duration, with an overall mean duration of 5.6 ± 3.6 years (p > 0.05). The frequency of genotypes did not differ in regards to sex or race (p > 0.05). The presence of the T allele compared with its absence was associated with a diagnosis of T2DM at a younger age (p = 0.047), lower neck/thigh ratio (p = 0.008), and a trend toward a smaller neck circumference (p = 0.053) (Table 1).

**Table 1:** Baseline characteristics of the overall cohort (*n *= 162)

Variable	rs7903146	Mean	SD	N	p values
Age (years)	CC	58.42	6.15	67	0.054
	CT/TT	56.26	7.96	95	
Age/diagnosis	CC	53.27	6.66	67	*0.047*
	CT/TT	50.88	8.47	94	
BMI	CC	30.43	4.51	67	0.823
	CT/TT	30.61	5.44	95	
Waist (cm)	CC	103.43	11.18	67	0.804
	CT/TT	102.99	10.92	94	
Neck (cm)	CC	38.69	3.43	67	0.053
	CT/TT	37.63	3.34	93	
Neck/thigh ratio	CC	0.67	0.08	67	*0.008*
	CT/TT	0.64	0.07	93	
Glucose (mg/dL)	CC	146.69	59.48	61	0.988
	CT/TT	146.84	58.63	91	
HbA1c (%)	CC	7.57	1.70	61	0.943
	CT/TT	7.59	1.78	87	
HDL cholesterol (mg/dL)	CC	46.48	10.88	60	*0.041*
	CT/TT	50.89	13.87	87	
Uric acid (mg/dL)	CC	5.93	1.78	53	*0.017*
	CT/TT	5.23	1.40	83	
Insulin (μIU/mL)	CC	17.65	12.13	63	0.099
	CT/TT	14.55	10.80	90	
C-peptide (ng/mL)	CC	3.47	1.65	63	0.015*
	CT/TT	2.87	1.36	93	

All analyses were performed with Student’s *t* test, with the exception of (*), which was performed with the Mann–Whitney test

Italic values indicate statistically significant associations

*SD* standard deviation, *BMI* body mass index, *HbA1c* glycosylated hemoglobin

At baseline, we observed differences in mean C-peptide values among the rs7903146 variants (p = 0.040), which were significantly higher in CC than CT (p = 0.029) and TT (p = 0.037) patients. Table 1 also shows that carriers of the T allele had lower mean values of uric acid (p = 0.017) and higher mean values of HDL-cholesterol (p = 0.041) than noncarriers of this allele. In the evaluation of the HOMA model using baseline glucose and C-peptide concentrations, T allele carriers presented greater insulin sensitivity (%S) (p = 0.021) and lower insulin resistance (p = 0.020) than noncarriers of this allele.

### Responses to the meal test before and after exenatide

Table 2 shows the results of laboratory parameters measured during the meal test before and 8 weeks after treatment with exenatide.

**Table 2:** Results of weight and laboratory parameters of C-Reactive Protein, tumor necrosis factor alpha, and glycosylated hemoglobin measured before and 8 weeks after treatment with exenatide in a subcohort of 56 patients with type 2 diabetes mellitus, subdivided according to *TCF7L2* genotype

Variable	Comparison	Standard error	p	IC (95%)	
				Inferior	Superior
Weight (kg)	CC (pre) VS CC (post)	0.04	*< 0.001*	2.08	2.25
	CC (pre) VS CT/TT (pre)	3.71	NS	− 7.51	7.05
	CC (post) VS CT/TT (post)	3.71	NS	− 7.19	7.37
	CT/TT (pre) VS CT/TT (post)	0.04	*< 0.001*	2.41	2.56
HbA1C (%)	Pre × post	0.11	*0.036*	0.02	0.46
CRP (mg/dL)	CC (pre) × CC (post)	0.33	NS	− 1.02	0.27
	CC (pre) × CT/TT (pre)	1.27	NS	− 4.18	0.81
	CC (post) × CT/TT (post)	1.15	NS	− 2.52	1.99
	CT/TT(pre) × CT/TT(post)	0.46	NS	0.15	1.94
TNFα (pg/mL)	CC × CT/TT	0.12	*< 0.001*	− 0.96	− 0.48
	Pre × post	0.04	*< 0.001*	0.23	0.41

Italic values indicate statistically significant associations

*pre* before treatment, *post* after treatment, *NS* non-significant

We evaluated 56 subjects prior to exenatide treatment (26 patients with the CC variant and 30 with the CT/TT variants) and 46 subjects after the exenatide treatment (21 CC and 25 CT/TT). Ten patients discontinued treatment, four of whom did so due to private reasons and six due to adverse effects of exenatide, mostly nausea, vomiting, and abdominal pain. Of the six patients who discontinued treatment due to adverse effects, there were three each with CC and TT genotypes, suggesting no imbalance of genotype on tolerability among these individuals.

### Plasma glucose curves

**Fig. 1:**
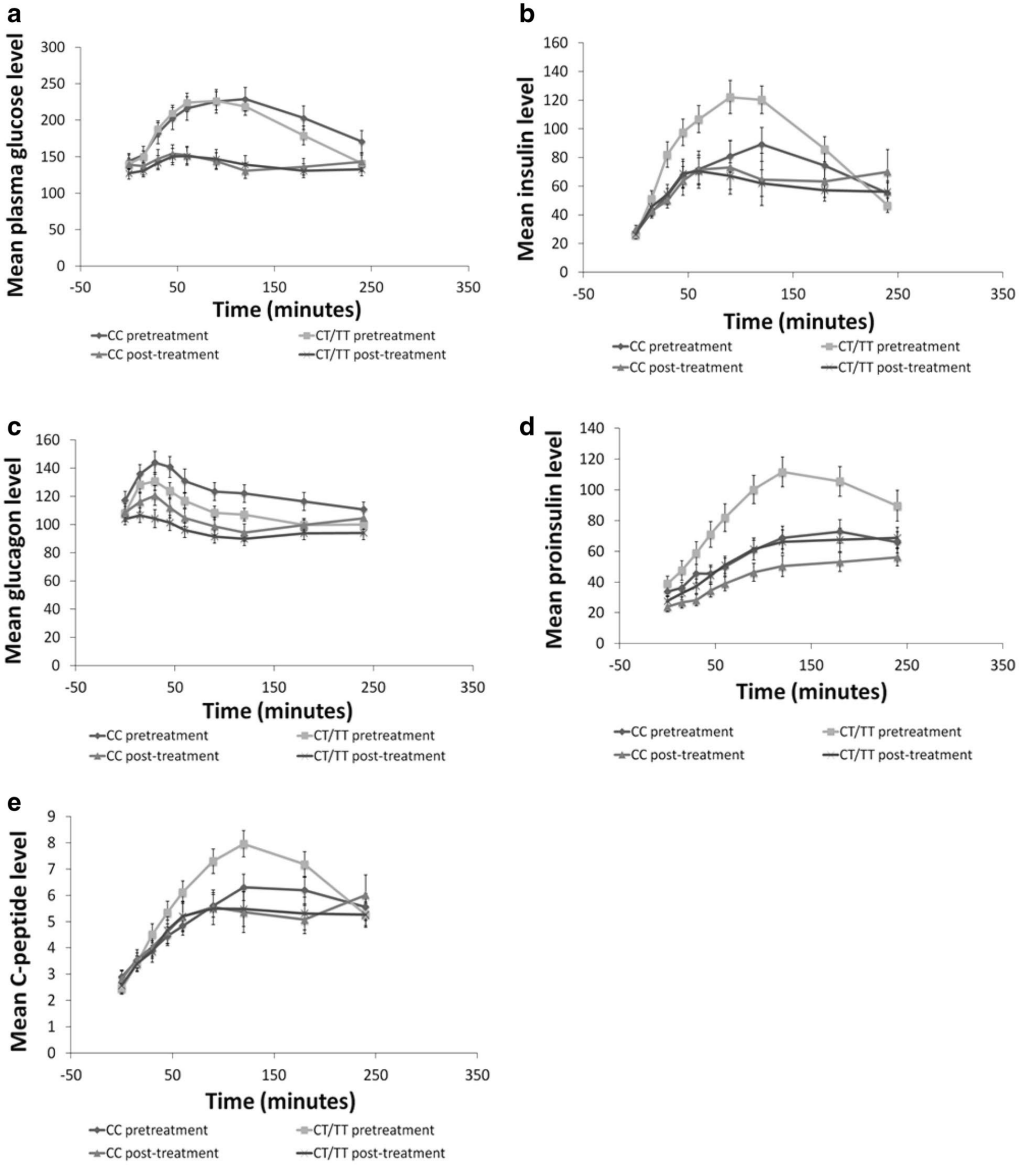
Plasma concentrations of **a** glucose, **b** insulin, **c** glucagon, **d** proinsulin, and **e** C-peptide levels during the meal test before and after treatment with exenatide for 8 weeks in patients with type 2 diabetes mellitus subdivided according to *TCF7L2* genotype

Plasma glucose values were similar in patients with CC and CT/TT genotypes during the meal tests before and after exenatide treatment. Both groups also presented a significant reduction (p = 0.003) in plasma glucose values after exenatide compared with values obtained before treatment. At 30–180 min, the mean glucose levels before treatment were higher than those after treatment in both groups (p < 0.05). Both groups presented similar areas under the glucose curve between 0 and 180 min before treatment and a similar decrease after treatment (p < 0.001) (Fig. 1a).

### Insulin

Before exenatide, insulin levels at 30–120 min were higher in CT/TT subjects compared with CC ones (p < 0.05). After treatment with exenatide, only CT/TT individuals demonstrated a significant insulin reduction at 30–180 min during the meal test compared with CC subjects (p < 0.05). Patients with the CC genotype presented no differences in insulin concentrations before and after treatment. The area under the insulin curve between 0 and 180 min was similar between groups before exenatide but decreased only in the CT/TT group after exenatide (p < 0.001) (Fig. 1b).

### Glucagon

Glucagon concentrations were similar in patients with CC and CT/TT genotypes before treatment and decreased at 15–120 min after treatment in both groups (p < 0.001). The areas under the glucagon curve between 0 and 180 min were similar before treatment and reduced after treatment in both groups (p < 0.001) (Fig. 1c).

### Proinsulin

Proinsulin levels at 45–240 min (p < 0.05) were higher in CT/TT subjects before exenatide and reduced only in this group after exenatide (p < 0.001). Although patients with the CC genotype also showed a reduction in proinsulin levels after treatment with exenatide, the reduction was lower than that observed in the CT/TT group. The area under the proinsulin curve was higher in the CT/TT group prior to treatment and decreased only in this group after treatment (p < 0.001) (Fig. 1d).

### C-peptide

The area under the C-peptide curve between 0 and 180 min was not significantly different between both genotype groups before exenatide and only decreased significantly after treatment in CT/TT (p < 0.001) (Fig. 1e).

### Weight, C-reactive protein, tumor necrosis factor alpha, and glycosylated hemoglobin

Patients with CC and CT/TT genotypes showed significant reductions in weight after treatment with exenatide (both p < 0.001), with mean decreases at 8 weeks of 2.16 ± 2.2 kg in CC patients and 2.49 ± 6.8 kg in CT/TT patients (p for comparison between groups = nonsignificant). Both groups showed comparable serum CRP levels before and after treatment. TNF-α concentrations also decreased after treatment with exenatide in both groups (p < 0.001); however, the levels were higher in the CT/TT group both before and after treatment, with a mean difference of 0.72 pg/mL in relation to the CC group (p < 0.001). Both groups had similar serum HbA1c levels before treatment and similar significant reductions after treatment (mean difference in both groups before and after treatment 0.24%, 95% confidence interval 0.02–0.46%, p = 0.036) (Table 2).

### Insulin secretion index (IGI 30) and homeostatic model assessment (HOMA Oxford)

There were no differences in IGI 30 between patients with CC and CT/TT genotypes before (p > 0.05) or after treatment (p > 0.05). In regards to HOMA, we observed that the estimated beta cell function (%B) was similar in both groups, but there was a trend toward lower values in carriers of the T allele (CT/TT patients) (p = 0.053). The estimated beta cell function (%B) increased after treatment in both groups (mean 21.3%, p = 0.021). Insulin resistance differed between the genotype groups (p = 0.042) before and after treatment, and was on average 0.87 lower in carriers of the T allele.

## Discussion

The results of our study show that the presence of the *TCF7L2* rs7903146 T allele in individuals with T2DM was associated with increased secretion of insulin, proinsulin, and C-peptide in response to a meal when compared with noncarriers of this allele, and no differences in serum glucagon or glucose concentrations. After treatment with exenatide, those carriers of the T allele showed a significantly decreased postprandial plasma insulin and C-peptide peak levels comparing with noncarriers. Both groups had a reduction in the proinsulin peak; however, the reduction was greater in patients with the T allele.

Impaired insulin secretion is recognized as one of the diabetogenic effects of *TCF7L2* [8, 34], and a negative effect on insulin sensitivity of the *TCF7L2* rs7903146 T allele has been demonstrated in previous studies [5, 15, 32]. In this population of subjects with T2DM, we observed that the group of T allele carriers had lower fasting plasma C-peptide concentrations than noncarriers of this allele, while all patients presented similar plasma glucose and insulin concentrations. However, we observed a higher elevation in postprandial insulin and proinsulin concentrations in patients with the T allele, a fact that differed from previous studies performed in nondiabetic subjects during an oral glucose tolerance test (OGTT) [11, 26].

In the results obtained with the HOMA model, we observed that carriers of the T allele compared with noncarriers of this allele had higher %S, lower IR, and a trend toward a decreased beta cell function in T allele carriers before treatment. We must emphasize that the HOMA model is based on baseline laboratory values and, in this study, we observed that the greatest differences between the genotypes were expressed during the meal test. In addition, noncarriers of the T allele showed some phenotypic characteristics associated with insulin resistance, such as greater neck/thigh ratio, lower HDL-cholesterol plasma concentrations, and higher uric acid concentrations.

After treatment with exenatide, both the CC and CT/TT groups showed reductions in serum levels of postprandial glucose. However, serum insulin concentrations, which before exenatide were higher during the meal test in carriers of the T allele, only reduced significantly after treatment in this group. Some studies have suggested that carriers of the T allele may present reduced insulin sensitivity combined with beta cell failure to compensate fully for the degree of resistance [4, 5, 15]. The lower elevations in postprandial insulin levels in T allele carriers observed in our study, associated with comparable blood glucose and similar weight reduction after treatment, suggest that treatment with GLP-1 mimetic promotes secretion of a more efficient insulin, improves insulin sensitivity, or even determines better uptake of glucose mediated by GLP-1 in CT/TT subjects compared with CC ones. This suggests that carriers of the T allele are in some aspects more responsive to treatment with GLP-1 agonists. On the other hand, treatment with exenatide was also effective in reducing postprandial serum glucagon concentrations independent of genotype, showing that the alpha cell response to GLP-1 is not affected by the *TCF7L2* genotype [30]. A recent study has shown that along with reduced insulin secretion, decreased glucagon suppression may contribute to the predisposition to diabetes conferred by *TCF7L2* [29]. We believe that the reduction in hepatic glucose production due to decreased glucagon may not have influenced the observed response. Furthermore, it is known that response to exenatide treatment may be related to other factors that improve insulin sensitivity, such as changes in inflammatory cytokines [3, 6]. In our study, we observed a reduction in TNF-α concentrations after treatment, regardless of the patient’s genotype. But T allele carriers maintained higher values; both before and after the use of the medication. Therefore, the antiinflammatory effect associated with exenatide may have contributed to the response observed.

Proinsulin concentrations were higher in carriers of T allele before treatment and showed a significant reduction in after treatment. The elevation in proinsulin is probably associated with a defect in beta cell processing or insulin resistance [22, 23, 31]. The greater reduction in proinsulin observed in the CT/TT group after treatment corroborates the hypothesis that the use of exenatide in these individuals may play a more important role in the functional response of the beta cell in patients with the T allele when compared with those without this allele. Thus, the results of the present study provide further evidence that the factors linking *TCF7L2* gene polymorphism to T2DM may lie in two of the major mechanisms of the pathophysiology of disease, namely, defects in GLP-1 mediated insulin secretion at a pancreatic level, and GLP-1 peripheral action. Therefore, the lower elevation in serum insulin and proinsulin levels observed in T allele carriers after exenatide may have occurred in part due to known extra-pancreatic effects of GLP-1 in the liver, skeletal muscle, and adipose tissue, improving the uptake of glucose by these tissues.

Although it is unclear whether GLP-1 receptors are expressed in peripheral tissues sensitive to insulin, some studies have suggested that treatment with GLP-1 agonists is associated with improved insulin sensitivity [22, 35]. Exenatide is believed to stimulate glucose transport and metabolism in the liver of rats, skeletal muscle, and adipose tissue in an insulin-independent manner (Gonzalez et al. 2005). In mice muscle, exenatide, like GLP-1, stimulates the in vivo expression of glucose transporters [2]. Therefore, the glucoregulatory GLP-1-like effects of exenatide are believed to also occur through its interaction with GLP-1 receptors in liver and muscle [2, 19], which appear to be structurally and/or functionally different from those in the pancreas. There is evidence that the phenotypic characteristics of T allele carriers, which include decreased glucose-stimulated insulin secretion and decreased hepatic insulin sensitivity, are similar to those observed in GLP-1 receptor knockout mice [14]. GLP-1 mimetic may contribute to a reduction in postprandial glucose by increasing muscle utilization and/or decreasing the hepatic production of glucose [9]. These characteristics are evident in our findings, which suggest that the action of the GLP-1 agonist, especially in T allele carriers, promoted a lower elevation in postprandial plasma concentrations of proinsulin, insulin, and C-peptide.

GLP-1 has also been shown to inhibit the secretion of glucagon, as well as the paracrine and inhibitory effects of insulin on glucagon secretion by the pancreatic alpha cell [13]. Other unknown mechanisms may regulate the function of these cells. Also considering that the postprandial hypoglycemic effect of exenatide has been explained by glucagon suppression and delayed gastric emptying [12], the effect of reducing glucagon after treatment was well demonstrated in the present study, and no differences according to genotypes were found regarding this variable.

We would like to point out some limitations of our study. First, owing to its design, the study did not include a placebo group; hence, the patients were not randomized to placebo or treatment arms. A control group was also not included; therefore, some comparisons were restricted to the results obtained before and after exenatide treatment. A second limitation was the lack of measurement of exenatide levels to assess patients’ compliance.

## Conclusion

In conclusion, the present study analyzed the response to exenatide according to rs7903146 polymorphism in patients with T2DM and provides additional insight on. Our findings suggest an improved effect of exenatide in carriers of this variant of the *TCF7L2* gene.
